# α‐Synuclein in Parkinson's Disease: From Bench to Bedside

**DOI:** 10.1002/med.22091

**Published:** 2024-12-20

**Authors:** Gabriele Bellini, Vanessa D'Antongiovanni, Giovanni Palermo, Luca Antonioli, Matteo Fornai, Roberto Ceravolo, Nunzia Bernardini, Pascal Derkinderen, Carolina Pellegrini

**Affiliations:** ^1^ Center for Neurodegenerative Diseases, Unit of Neurology, Parkinson's Disease and Movement Disorders, Department of Clinical and Experimental Medicine University of Pisa Pisa Italy; ^2^ Department of Neurology The Marlene and Paolo Fresco Institute for Parkinson's and Movement Disorders, NYU Langone Health New York City New York USA; ^3^ Unit of Histology and Embryology, Department of Clinical and Experimental Medicine University of Pisa Pisa Italy; ^4^ Unit of Pharmacology and Pharmacovigilance, Department of Clinical and Experimental Medicine University of Pisa Pisa Italy; ^5^ Department of Neurology Nantes Université, CHU Nantes, INSERM Nantes France

**Keywords:** α‐synuclein, central nervous system, clinical trials, gut‐brain axis, immune cells, neurodegeneration, neuroinflammation, Parkinson's disease, preclinical evidence, spreading

## Abstract

α‐Synuclein (α‐syn), a pathological hallmark of PD, is emerging as a bridging element at the crossroads between neuro/immune‐inflammatory responses and neurodegeneration in PD. Several evidence show that pathological α‐syn accumulates in neuronal and non‐neuronal cells (i.e., neurons, microglia, macrophages, skin cells, and intestinal cells) in central and peripheral tissues since the prodromal phase of the disease, contributing to brain pathology. Indeed, pathological α‐syn deposition can promote neurogenic/immune‐inflammatory responses that contribute to systemic and central neuroinflammation associated with PD. After providing an overview of the structure and functions of physiological α‐syn as well as its pathological forms, we review current studies about the role of neuronal and non‐neuronal α‐syn at the crossroads between neuroinflammation and neurodegeneration in PD. In addition, we provide an overview of the correlation between the accumulation of α‐syn in central and peripheral tissues and PD, related symptoms, and neuroinflammation. Special attention was paid to discussing whether targeting α‐syn can represent a suitable therapeutical approach for PD.

## 1 Introduction

Growing evidence highlights that neuroinflammatory processes contribute to the pathogenesis of Parkinson's disease (PD) [1]. In this setting, α‐synuclein (α‐syn), a presynaptic neuronal protein that is linked genetically and neuropathologically to PD, is emerging as a bridging element between the trigger of neuro/immune‐inflammatory responses and the progression of neurodegeneration in PD [2]. Accumulation of misfolded and aggregated α‐syn in neurons and non‐neuronal cells of the brain and peripheral autonomic networks, as well as in circulating exosomes and gut, can compromise synaptic transmissions, act as prion‐like protein contributing to disease spreading and trigger neurogenic/inflammatory responses in PD [3, 4]. For instance, intestinal pathological α‐syn injection in mice was associated with gut‐to‐brain spread of α‐synucleinopathy and associated neurodegeneration and behavioral deficits via vagal transmission [5]. In parallel, pathological α‐syn might result in robust activation of immune and inflammatory cells that, in turn, may contribute to systemic and central neuroinflammation associated with PD [1, 6]. Of interest, recent and pioneering evidence suggests that the initial localization of pathological α‐syn forms can also identify specific PD phenotypes; α‐syn accumulation primarily in the brain appears to identify a “brain first” PD phenotype, while an early α‐syn deposition in the gut identifies a “body first” PD phenotype characterized by early autonomic dysfunction and REM sleep behavior disorder (RBD) during the prodromal phase [7].

On these bases, the present review provides a comprehensive overview of the available knowledge on the physiological and pathological forms of α‐syn and critically discusses, covering clinical and basic research aspects, how central and systemic α‐syn deposition is at the crossroads between neuroinflammation and neurodegeneration in PD. In addition, we review clinical evidence showing a correlation between the accumulation of α‐syn in central and peripheral tissues and PD diagnosis, duration, motor and non‐motor symptoms, neurodegeneration, and neuroinflammation. Special attention was also paid to discuss whether current pharmacological strategies aimed at counteracting α‐syn can represent a suitable therapeutical approach for PD.

## 2 Physiological and Pathological Forms of α‐Syn

α‐Syn was first isolated in 1988 from the Torpedo electric organ and named after its localization on synaptic vesicles and nuclear envelopes [8]. The human 140‐aminoacid homolog was identified a few years later in the brain [9]. These first observations on α‐syn found relatively little echo until 1997 when point mutations in the six‐exon gene that encodes α‐syn (*SNCA*) mapping to chromosome 4q21 were identified as a rare cause of autosomal‐dominant PD [10]. The same year, it was shown that Lewy bodies (LBs) and neurites (LNs), the pathological hallmarks of PD, were highly immunoreactive for α‐syn in sporadic PD brains, thereby suggesting that α‐syn was one of the main components of these intraneuronal inclusions [11].

**Figure 1:**
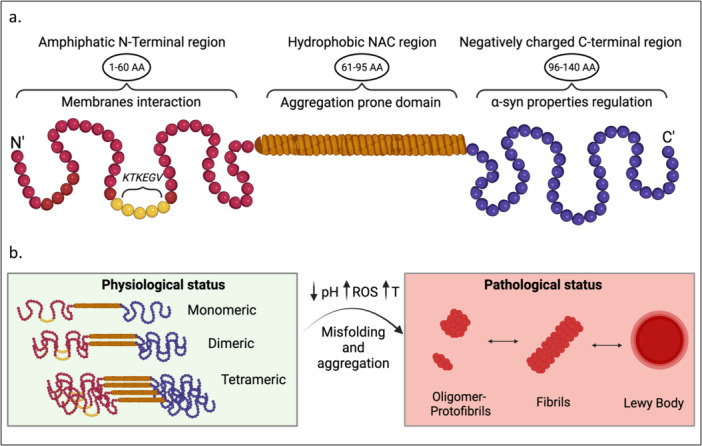
Schematic illustration of α‐synuclein pathway (a) α‐syn structure: 1. the amphipathic N‐terminal region (red), implicated in interactions with membranes. It contains the highly conserved 11‐residue repeats‐KTKEGV (yellow); 2. The hydrophobic NAC region (orange), has a major role in aggregation; 3. The negatively charged C‐terminal region (purple), regulates α‐syn interactions with other ligands. (b) In physiological status (green), α‐syn can be present through unstructured soluble monomeric and tetrameric forms. In pathological status (red), α‐syn aggregates into oligomers‐protofibrils, and fibrils, resulting in the development of Lewy Body. Abbreviations: AA, amino acids; α‐syn, α‐synunclein; T, temperature. [Color figure can be viewed at wileyonlinelibrary.com]

Since 1997, a large body of work focused on the physiological and pathological roles of α‐syn. α‐Syn is composed of three biochemically distinct domains: (i) a highly conserved and positively charged amino‐terminal repeat (residues 1–60) showing similarity with apolipoproteins, which allows the protein to fold into an amphipathic α‐helix when it is bound to lipid membranes or in the presence of phospholipids [12] (ii) a central hydrophobic region (amino acids 61–95) known as non‐amyloid component (NAC), which contains a 12‐aminoacid stretch that is necessary and sufficient to form the proteolysis‐resistant fibril core [13], and (iii) a C‐terminal acidic tail (amino acids 96–140) accountable for most interactions with other proteins and small molecules that contains most of the phosphorylation sites and especially serine 129 (pS129) (Figure 1). The exact physiological functions of α‐syn are still elusive, but its association with the distal reserve pool of synaptic vesicles and the deficiencies in synaptic transmissions observed in response to overexpression or suppression of the *SNCA* gene suggest that it plays a role in the release of neurotransmitter and synaptic homeostasis, thereby influencing neuronal plasticity and survival [14]. Recent evidence also suggests that low levels of α‐syn in the nucleus of neuronal cells can influence cell transcription [15].

As a typical intrinsically disordered protein, α‐syn adopts a monomeric, random coil conformation under physiological conditions [16]. It has also been suggested that physiological α‐syn occurs as a stable α‐helically‐folded tetramer that is resistant to amyloid formation [17], but these findings are still highly debated [18]. α‐syn has a natural propensity to aggregate, and this phenomenon is strongly influenced by the cellular environment [19]. For instance, lowering of pH, increasing of temperature, hormonal changes, and oxidative stress, together with increases in hydrophobicity or decreases in charge, can modify α‐syn conformation, thereby influencing its misfolding and aggregation [20, 21, 22]. These processes involve the formation of non‐fibrillar off‐pathway and soluble transient pre‐fibrillar intermediate species, regarded as oligomers and protofibrils, which eventually convert into insoluble fibrillar aggregates with distinct cross β‐sheet conformation [16]: these two species seem to be more neurotoxic than mature fibrils due to their instability and smaller size [23, 24]. The increase in wild‐type α‐syn or mutated α‐syn can promote intracellular accumulation and accelerate the fibrillation process via a nucleation‐dependent mechanism [25]. The long‐term consequences of β‐strand α‐syn aggregates are the disruption of neuronal functions with consequent neuronal death [26]. Specifically, overexpression of α‐syn inhibits endoplasmic reticulum (ER)‐Golgi trafficking and Golgi fragmentation, impairs mitochondrial function, blocks ubiquitin‐proteasome system, and alters the lysosomal system [27, 28]. Abnormal or increased α‐syn has also been associated with suppression of exo/endocytosis processes, altering the distribution of synaptic Soluble N‐ethylmaleimide‐sensitive factor activating protein receptor (SNARE) proteins and influencing the activity of other peripheral proteins such as complexins and synapsins [29, 30, 31, 32, 33, 34]. In addition to the effects of the microenvironment, α‐syn post‐translational modifications also have a major impact on protein conformation. A very large number of α‐syn post‐translational modifications have been identified so far, but the most frequent and by far the most studied is phosphorylation at serine 129. While, under physiological conditions, only a small proportion of α‐syn is phosphorylated (estimated around 4%) [35], more than 90% of α‐syn is phosphorylated on this residue, suggesting a role for pS129 in inclusion formation [35, 36].

Although initially considered as being purely intracellular, it is now well demonstrated that α‐syn can be secreted by cells. Several mechanisms have been proposed to explain how cytosolic α‐syn, either monomeric or aggregated, is conveyed into the extracellular milieu, including unconventional exocytosis [37, 38], exosomes [39, 40], and membrane nanotubes [41]. These findings were corroborated in vivo by showing that both monomeric and oligomeric α‐syn could be detected in the blood and cerebrospinal fluid (CSF) of healthy and PD subjects [42, 43]. The secretion of α‐syn aggregates from neuronal cells can depend on various stress cellular conditions (e.g., proteasomal and mitochondrial dysfunction, unconventional ER/Golgi‐independent exocytosis) or dopamine that, besides increasing the formation of non‐fibrillar α‐syn oligomers, enhances the secretion process [44]. Once dispersed in the extracellular milieu, α‐syn can be passively or endocytically internalized by other neuronal, glial, and immune/inflammatory cells, and a few receptors have been so far identified which can facilitate such a process, including heparan sulfate proteoglycans (HSPGs) [44] and toll‐like receptor 2 (TLR2) [45]. A role for the lymphocyte activation gene 3 (LAG3) has also been suggested [46], but these findings are still debated [47]. All these receptors preferentially bind α‐syn in the amyloid over the monomeric state to initiate cell‐to‐cell transmission and/or to activate inflammatory response in microglia [44, 46, 47], while monomeric‐syn seems to be predominantly internalized via a passive mechanism [48]. Several in vitro studies showed that once internalized, extracellular misfolded α‐syn, either oligomeric or in the form of preformed fibrils, is capable of promoting the recruitment of endogenous soluble α‐syn into insoluble deposits in a mechanism reminiscent of the nucleation capacity of prions [49, 50]. Following these in vitro findings, the transmission and propagation of α‐syn along neuronal connections have been observed after injection of pathological α‐syn (either obtained from diseased brain homogenates or by generating preformed fibrils from recombinant protein) in the brain or peripheral autonomic nervous systems of rodents and non‐human primates [3, 51, 52, 53, 54].

Taken as a whole, these results show that α‐syn can transmit the aggregation process between cells and tissues and, therefore, confirm that it could behave like a prion or at least like a propagon [55]. They might also explain the presence of α‐syn–positive inclusions in grafted neurons in PD subjects who had received fetal neuron transplants [56, 57], and they provide a molecular basis to the spreading of PD pathology in specific stages as proposed initially by Braak and more recently by Borghammer [7, 58].

Of interest, there is evidence that peripheral α‐syn can spread and contribute to brain pathology [2, 59]. Specifically, peripheral infusion of α‐syn seeds may induce central nervous system (CNS) α‐syn pathology. Various mechanisms have been proposed to explain CNS neuroinvasion, but retrograde axonal transport (e.g., through the vagal nerve) is thought to be the main pathway advancing α‐syn pathology via anatomically interconnected pathways of susceptible cell populations [59]. Indeed, the propagation of α‐syn is aligned with the stereotypical pattern of progressive LB as proposed by Braak and colleagues and with the appearance of clinical signs in the disease course [58, 60]. Indeed, in most cases of PD, the pathological process has been hypothesized to start with the α‐syn accumulation in the olfactory structures or the gastrointestinal (GI) tract and, subsequently, to spread in a trans‐synaptic prion‐like fashion in the CNS, contributing to brain pathology [61]. Supporting this view, subdiaphragmatic vagotomy has been found to prevent the spread of misfolded α‐syn from the gut to substantia Nigra pars compacta (SNpc) as well as the occurrence of parkinsonism in an environmental animal model following injections of α‐syn preformed fibrils in the gut [5, 62]. Moreover, the injection of human α‐syn into the rat nodose ganglion spread to gastric vagal endings as well as to the dorsal motor nucleus of the vagus nerve [63]. A recent study has also reported that the centripetal spreading of misfolded α‐syn could also be mediated by enteroendocrine cells lining the GI tract [64].

Based on this body of evidence, it has been hypothesized that the initial localization of pathological α‐syn forms can also identify specific PD subtypes. In particular, α‐syn accumulation primarily in the brain appears to identify a “brain first” PD phenotype, while an early α‐syn deposition in the gut identifies a “body first” PD phenotype characterized by early autonomic dysfunction and RBD [7, 65, 66].

## 3 Neuronal and Non‐Neuronal α‐Syn Across Neuroinflammation and Neurodegeneration

**Figure 2:**
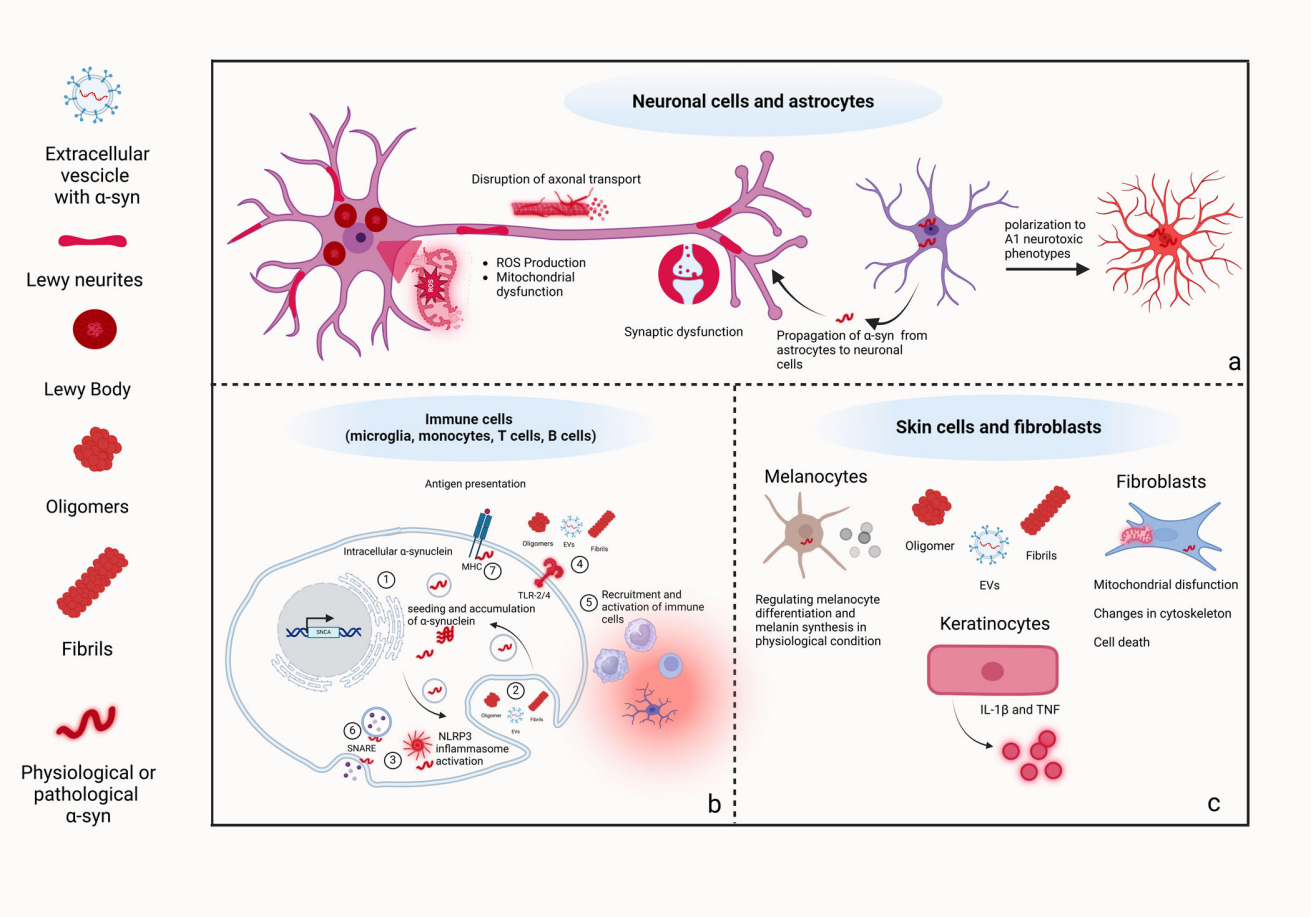
Schematic representation of the effects of α‐synuclein on neuronal and non‐neuronal cells. (a) Neurons: α‐syn aggregates, Lewy bodies or Lewy neurites, can promote ROS formation, alter cellular components, including proteins and DNA, and induce mitochondrial dysfunction, with consequent axonal transport disruption and synaptic dysfunction. In parallel, pathological α‐syn can trigger the polarization of astrocytes to neurotoxic A1 phenotype, leading to the release of ROS and pro‐inflammatory cytokines in the extracellular space. (b) Immune/inflammatory cells: α‐syn can be produced within immune cells that express the SNCA gene (1) or its pathological forms can be internalized from the extracellular space via endocytosis (2). Once in the intracellular space, α‐syn can seed and accumulate, forming oligomers, fibrils, and aggregates, triggering inflammatory pathways, including NLRP3 inflammasome signaling (5). Of note, both pathological and physiological forms of α‐syn can interact with TLR‐2/4 (4), promoting cellular polarization towards a pro‐inflammatory phenotype as well as the recruitment and activation of other immune/inflammatory cells (5). α‐syn can also interfere with MHC and SNARE complexes, thereby affecting antigen presentation and the trafficking of cell‐surface receptors (6, 7). (c) Physiological α‐syn in melanocytes promotes the melanin‐producing process. In keratinocytes, oligomeric α‐syn induces the release of pro‐inflammatory cytokines, such as IL‐1β and TNF. Additionally, α‐syn can be internalized by fibroblasts, leading to increased mitochondrial dysfunction, alterations in the cytoskeleton, and in turn, fibroblast cell death. Abbreviations: α‐syn, alpha‐synuclein; IL‐1β, interleukin‐1β; MHC, major histocompatibility complex; NLRP3, Nod‐like receptor protein type 3; ROS, reactive oxygen species; SNARE, soluble N‐ethylmaleimide‐sensitive‐factor attachment protein receptor; TNF, tumor necrosis factor; TLR2/4, toll‐like receptor 2 and 4. [Color figure can be viewed at wileyonlinelibrary.com]

**Figure 3:**
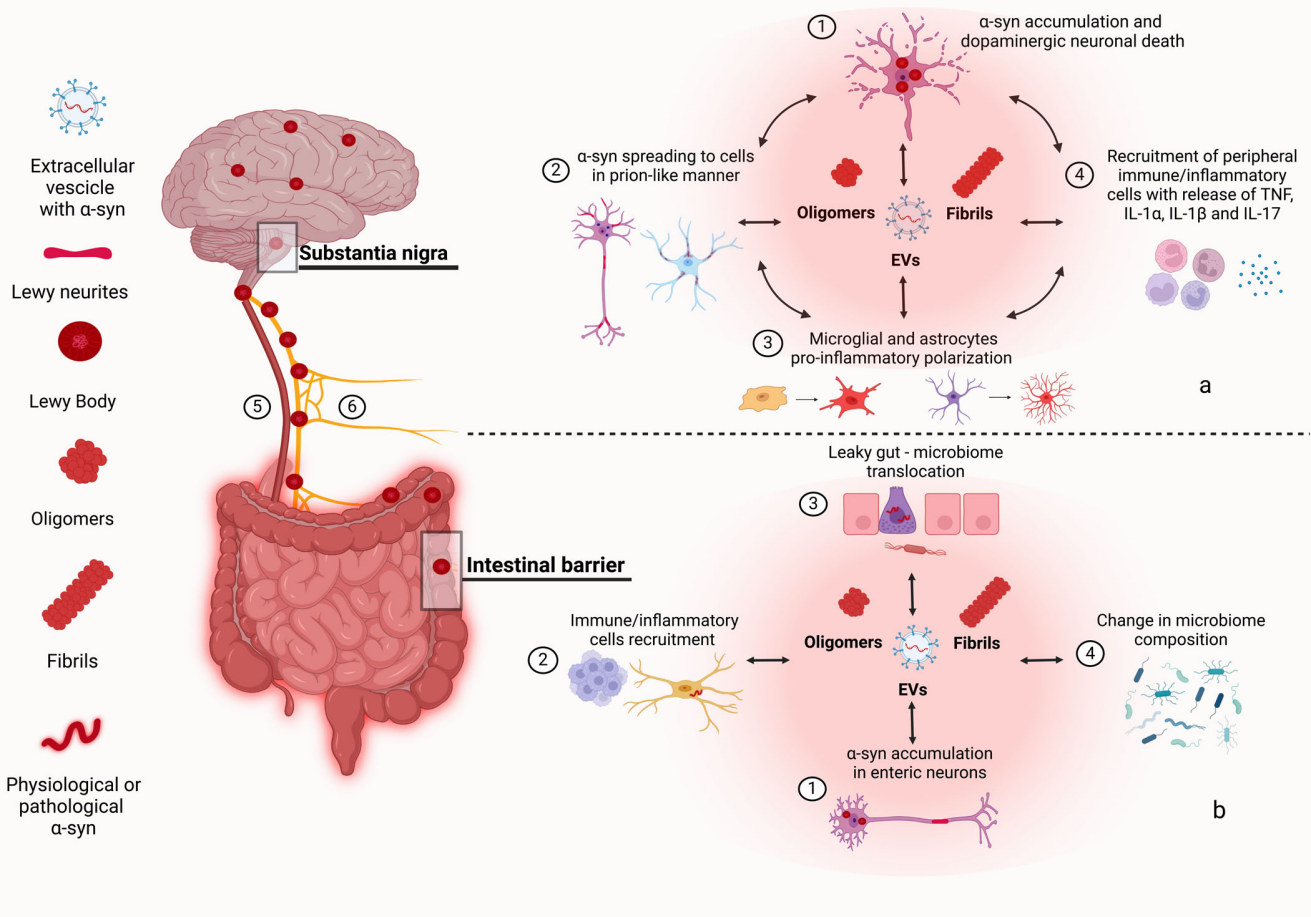
Schematic representation of the interplay between α‐synuclein, neuroinflammation, and neurodegeneration in Parkinson's disease (a) Within the CNS, pathological α‐syn species are found in neuronal and non‐neuronal cells, including dopaminergic neurons in the su*bstantia nigra*, and microglial and astroglial cells, respectively, as well as in the extracellular space (1, 2, 3). The intra‐ and extracellular α‐syn promotes neuronal death (1), spreads, in a prion‐like manner, among neuronal cells (2), and induces microglial and astroglial activation (3), that, besides contributing to neurogenic/inflammatory responses, promotes the recruitment of peripheral immune/inflammatory cells (4), that further amplifying the ongoing neurodegenerative and neuroinflammatory processes. (b) The accumulation of pathological α‐syn in the ENS (1), in immune cells (2) as well as in gut barrier cells (3) can promote neurogenic/inflammatory responses, compromise further intestinal barrier integrity and permeability, and favor changes in microbiota composition (4). These events can contribute to the translocation of pathogens in the bloodstreams and α‐syn forms, including in EVs, that, in turn, can spread in the CNS via systemic circulation (5). In parallel, enteric α‐syn can spread in the brain via nerve ascending pathways (6). Both hypothesized spreading mechanisms contribute to neurodegeneration and neuroinflammation in the CNS. Abbreviations: α‐syn, alpha‐synunclein; CNS, central nervous system; ENS, enteric nervous system. [Color figure can be viewed at wileyonlinelibrary.com]

α‐syn is expressed in neuronal and non‐neuronal cell types, including central and enteric neurons, astrocytes, microglia, monocytes, macrophages, dendritic cells, neutrophils, NK cells, B and T cells, enterochromaffin cells (ECs) and skin cells, and its pathological forms can influence cell functions [67, 68, 69, 70, 71, 72]. In particular, oligomeric, aggregated, fibrils, and phosphorylated α‐syn forms can contribute to neuronal degeneration, activation of immune/inflammatory cell pathways, alterations of cell morphology and motility, impairments of gut barrier, and skin remodeling, degeneration and inflammation (Figure 2). Of note, several studies have focused on the characterization of the role of α‐syn in promoting central and peripheral neuroinflammatory and neurodegenerative processes that can contribute to PD pathology. Thus, based on the recent and pioneering brain‐first and body‐first PD hypothesis, the most prominent data on the role of central and enteric neuronal and non‐neuronal α‐syn in PD pathophysiology are addressed in the following sections and represented in Figure 3.

### 3.1 α‐Syn‐Driven Brain First Subtype

Pathological α‐syn forms in the neuronal and non‐neuronal cells of the CNS have been found to induce neuronal degeneration and synaptic dysfunction through mitochondrial dysfunction, lysosomal impairment, and membrane disturbances as well as neurogenic/inflammatory responses, respectively, that can contribute to PD onset and progression. For instance, α‐syn accumulation in central immune/inflammatory cells, including microglia, can activate inflammatory signaling as well as promote T cell recruitment and maturation towards pro‐inflammatory phenotype by interfering with SNARE complexes, altering the trafficking of cell‐surface receptors, or interacting with TLRs and NLRP3 inflammasome (Figure 2) [67, 73, 74, 75, 76, 77, 78]. In parallel, intracellular pathological α‐syn can be also secreted in extracellular space and, in turn, activate neighboring neuronal and non‐neuronal cells, thus creating a cell microenvironment, that, continuously fed by α‐syn, contributes to the chronicization of the ongoing neuroinflammatory and neurodegenerative processes. Thus, α‐syn acts as a bridging element between neuroinflammation and neurodegeneration (Figure 3a) [71, 79, 80]. Indeed, clinical studies have documented concomitance of α‐syn inclusions, neuronal death, microgliosis, astrogliosis, and peripheral immune cell infiltration, mainly T cells (CD4^+^, CD8^+^, and CD3^+^ T cells) and monocytes, in PD post‐mortem brain sections [81, 82, 83, 84, 85, 86, 87, 88]. However, these findings from post‐mortem patients do not establish a causal relationship between α‐syn pathology, microgliosis, astrogliosis, and immune/inflammatory cell infiltration and the clinical stage of PD.

Studies performed in animal models have allowed to better understand the putative role of α‐syn as a bridging plug across neuroinflammation and neurodegeneration in PD. A recent pioneering study performed in a mouse model with selective microglial‐α‐syn overexpression in the *substantia nigra* (CX3CR1‐SNCA mice) showed that α‐syn deposition in microglial cells induced selectively dopamine neuronal degeneration by promoting phagocytic exhaustion and the recruitment of peripheral immune/inflammatory cells [89]. In particular, the authors showed that α‐syn accumulation in microglia altered oxidative stress‐related genes, increasing intracellular and extracellular ROS levels and NO production with consequent neurotoxicity and dopamine neuronal cell loss [89]. In addition, glial‐derived pro‐inflammatory mediators, recruited peripheral adaptive immune cells (i.e., T cells, dendritic cells, and B cells) [89, 90]. They, once recruited, can be activated by microglial cells [91]. Such events trigger a vicious cycle, that further induces the recruitment of peripheral leukocytes and microglial neurotoxicity. In accordance, CX3CR1‐SNCA nigral tissues were found enriched in numerous populations of innate and adaptive immune cells, including CD8+ and CD4 Th1+ T and NK cells that originated in the periphery and infiltrated brain tissues contributing to neuroinflammation and ongoing neurodegenerative process [45, 89, 90, 92, 93]. These findings suggest that intra‐ and extracellular α‐syn deposition in brain tissues can represent an early event in PD that contributes to dopaminergic neuronal death and inflammation by recruitment of peripheral immune/inflammatory cells. In support of this view, different studies performed in α‐syn‐driven animal models of PD, including transgenic mice and rats injected with pre‐formed α‐syn fibrils into the striatum, reported that microgliosis preceded neuronal death and that, infiltrating CD8^+^ T and MHC‐II cells were in close proximity to neuronal and non‐neuronal cells overexpressing α‐syn (Figure 3a) [94, 95, 96].

Regarding the astrocytes, an interesting study using human brain homogenates from PD patients reported that α‐syn is recruited and propagated from astrocytes to neurons, eliciting neural cell damage [97]. Supporting this view, a direct correlation between the pathological α‐syn accumulation in astrocytes and neurotoxicity has been shown using primary culture of astrocytes overexpressing α‐syn derived from A53T mice and from pluripotent stem cells from familial PD patients [98, 99]. In particular, pathological α‐syn promoted the polarization of astrocytes to neurotoxic A1 phenotype, leading to the release of ROS and pro‐inflammatory cytokines in the extracellular space (Figure 3a) [100]. Accordingly, Yun et al. [101], have demonstrated that the pharmacological blockage of astrocyte conversation to a neurotoxic A1 phenotype by NLY01 (glucagon‐like peptide agonist) in a mouse model of sporadic PD prevented central inflammation and neuronal death, prolonged survival and partially rescue of behavioral and neurological deficits.

### 3.2 α‐Syn‐Driven Body First Subtype

Of interest, the involvement of the gut‐brain axis in PD has fostered research on the characterization of the putative role of α‐syn accumulation in neuronal and non‐neuronal GI tract cells [102]. Using total and phospho‐α‐syn immunohistochemical staining, several independent neuropathology laboratories demonstrated that LBs and LNs‐like structures were observed in the enteric neurons in both the myenteric and submucosal plexus in the vast majority of PD patients [103, 104]. In addition, pathological α‐syn species, including aggregated [96], hyperphosphorylated [72], and truncated forms [97] with a seeding activity [105, 106] are found in the enteric neurons in PD, thereby recapitulating the key features of pathological α‐syn observed in the brain [36]. Of note, though physiological α‐syn has also been detected in ECs, there are no existing studies on the presence of pathological α‐syn deposits in the ECs in the gut of PD subjects.

Of interest, enteric α‐syn accumulation can also promote neurogenic/immune‐inflammatory responses that, in turn, impair intestinal barrier integrity and permeability or directly decrease tight junction protein expression [86, 107, 108]. Increased intestinal permeability allows the passage of α‐syn, either free or enclosed in extracellular vesicles (EVs), along with toxins, bacteria, and other molecules, into the systemic circulation. This process can promote the spread of α‐syn to the brain and activate systemic immune‐inflammatory cells thereby contributing to PD pathology [109, 110]. Supporting this view, recent studies in transgenic PD mice demonstrated that enteric α‐syn accumulation, mainly in myenteric and submucosal neurons, bowel inflammation, and alterations of intestinal barrier integrity and permeability occurred in the presymptomatic stages of the disease before the onset of central pathology (Figure 3b) [107, 111]. However, whether α‐syn deposition occurred also in other GI cell types, including enteric glia, epithelial cells, and immune/inflammatory cells has not been evaluated and, most importantly, a direct and mutual relationship among α‐syn accumulation, enteric inflammation, gut barrier impairments, and PD pathology has not been established. Studies in vitro have demonstrated that α‐syn incubation in macrophages promoted the direct activation of canonical caspase‐1‐dependent inflammasome signaling and IL‐1β release, which, in turn, contributed to impaired intestinal epithelial barrier integrity [107].

Of note, α‐syn can interact with gut microbiota, promoting changes in bacteria composition and function that could contribute to gut barrier alterations and enteric inflammation observed in PD [102]. In parallel, gut microbiota‐derived factors can influence α‐syn aggregation, thus generating a vicious circle that contributes to the progression of α‐syn pathology in the gut [102, 107, 112, 113]. However, a better understanding of the mechanisms by which α‐syn impacts gut cells is crucial for unraveling the link between α‐syn, GI symptoms and PD pathology.

## 4 α‐Syn Deposition and PD Pathogenesis

### 4.1 Central Nervous System

α‐Syn is constitutively expressed in the CNS, comprising 1% of total cytosolic proteins [114]. Previous immunohistochemical analysis in normal brain tissues showed that α‐syn is mainly localized in close proximity to presynaptic terminals, while neuronal soma staining is less apparent [115]. α‐Syn was also detected in the glial cytoplasm of human brains of healthy subjects [116]. Of note, the expression of α‐syn is influenced during neuronal development, following the determination of neuronal phenotype and in the establishment of synaptic connections [26]. Notably, physiological α‐syn was not detected in the primary visual and cerebellar cortex, regarded as brain areas not prone to develop α‐syn pathology [117]. Several previous studies have reported the presence of LBs in various sites of the CNS, including the hypothalamus, nucleus basalis of Meynert, substantia nigra, locus ceruleus, dorsal raphe nucleus, dorsal motor nucleus of the vagus nerve, intermediolateral nucleus of the spinal cord, and sacral autonomic nucleus [58, 118, 119, 120, 121]. LBs are also detected in neurons of the amygdala and the cerebral cortex, particularly in deep layers (V and VI) of the limbic system [122, 123]. Of note, LBs formation has been found to advance in a caudal to rostral manner from the brainstem to the cortex over the course of the disease (Braak staging system) [58]. However, it's important to note that not all cases of α‐synucleinopathy reflect the proposed Braak staging system [124]. More recent comparative analyses of healthy and PD‐affected brains have revealed the presence of α‐syn aggregates [58, 123, 124, 125]. In addition, the study conducted by Beach et al. demonstrated a significant correlation between LBs and motor symptoms assessed by unified Parkinson's disease rating scale (UPDRS) scores, as well as cognitive function measured by mini‐mental state examination (MMSE) scores, particularly in subjects with smaller fractions of coexisting Alzheimer's disease pathology [126]. Others have also shown that the quantity of LBs in the frontal and/or limbic cortex serves as a better predictor of dementia in PD [127, 128, 129]. However, some studies have reported that around 10% of asymptomatic individuals over 60 years old exhibited extensive LB pathology without any signs of clinical parkinsonism [130, 131]. In addition, Zaccai et al. reported an α‐syn‐related pathology in aged individuals without a history of parkinsonism, suggesting no correlation between α‐syn deposition and PD progression [132]. These conflicting results about the accumulation of α‐syn in the brain of PD patients could depend on different clinical features of recruited patients (e.g., disease duration, motor/non‐motor symptoms).

Two recent studies, aimed at evaluating α‐syn deposition in brain tissues from post‐mortem PD patients, have allowed us to better understand the spreading of pathological α‐syn in PD [133, 134]. In particular, the study from Borghammer et al. showed that α‐syn deposition in the olfactory bulb (OB) correlated with α‐syn accumulation in the amygdala, while PD patients with protein deposition in the brainstem or peripheral nervous system (PNS) showed less frequent α‐syn in OB. This pattern suggests two distinct origins of PD (PNS and OB), that rarely are involved simultaneously [133]. The other study from Lin et al. reported that microstructural alterations measured by diffusion magnetic resonance imaging (MRI) were associated with dopaminergic neuronal loss and LNs load in the SNpc, suggesting MRI as a suitable monitoring indicator for Lewy pathology [134].

Of interest, research on genetic PD patients has provided valuable insights. In particular, some patients with the A53T mutation in the SNCA gene (a missense point mutation), exhibited diffuse α‐syn deposition associated with specific clinical features (Table 1) [135, 136, 137].

**Table 1:** Central α‐syn/LBs detection in PD patients.

Study	Number of patients/HC	Demographic information PD (mean or median ± SD or range)		Disease characteristics PD (mean or median ± SD or range)		Sites of investigation	α‐Syn/LBs deposition	To note
		Age (years)	M/F	Disease duration (years)	Other			
Mattila 2000 [129]	Autopsy 45 PD	5.8 ± 6.2	22/23	1.8 ± 4.7	—	Amygdala, hippocampus and six cortical gyri	At least one cortical LB was found in 95% of cases.	..
Braak 2003 [58]	Autopsy 41 PD 69 without PD diagnosis but eith LNs/LBs in a subset of neuronal types 58 HC	75.7 ± 7.2	22/19	—	—	Braak'stages site: 1‐ DMNV 2‐ Medulla oblongata and Pontin tegmentum 3‐ Midbrain 4‐ Basal prosencephalon and mesocortex 5‐ Neocortex 6‐ Neocortex	100% in PD and in incidental case. 21 = Medulla oblungata 13 = medulla oblungata and pontine tegmentum 24 = midbrain 24 = basal prosencephalon and mesocortex 17 = sensory association areas of the neocortex and prefrontal neocortex 11 = sensory association areas of the neocortex, premotor areas, primary sensory areas and primary motor field	α‐syn deposition indicates a caudo‐rostral progression of brain pathology
Colosimo 2003 [138]	Autopsy 17 PD 21 PDD	Age at death PD = 75.4 (range: 51–91) PDD = 75.3 (range: 57–86)	PD 11/6 PDD 13/8	PD = 15.1 (range: 5–28) PDD = 15.5 (range: 8–27)	—	Neocortex and limbic system	PD Limbic pattern = 9/17 Neocortical pattern = 8/17 PDD Limbic pattern = 7/21 Neocortical pattern = 14/21	No correlation with cognitive impairment
Koväri 2003 [128]	Autopsy 22 PD	79.5 ± 1.6 years	11/11	4.8 ± 1.3	Parkinsonism preceded cognitive decline by at least 3 years	Brodmann areas 9, 21, 24, 40 and entorhinal cortex	Highest LB densities in the anterior cingulate and entorhinal cortex, only mild LBs involvement in the frontal cortex.	Correlation with CDR score
Jellinger 2004 [124]	Autopsy 116 AD, 71 PD, 38 DLB, 8 PSP, 1 senile tremor 26 HC	80.9 ± 7.9	36/35	8.9 ± 6.3	23% developed dementia	Neocortical areas (frontal, temporal, parietal, occipital), hippocampus parahippocampal area and superior temporal lobe, basal ganglia, midbrain, pons, medulla, and cerebellum	% α‐syn deposition in PD patients: 100% medullary, pontine and mesencephalic nuclei, 90.1% nucleus basalis, 58.9% limbic cortex, 46% cingulate cortex, 30.2% amygdala, 36.2% CA 2–3 hippocampal region, 28.8% neocortex, 11% striatum.	Confirm Braak's staging of LB‐pathology in PD
Croisier 2005 [139]	Autopsy 37 PD	78.7 ± 4.9 (All data available for 17 patients)	12/5	6.2 ± 12.1	—	SN	100% positive for α‐syn	Correlation with MHC II‐expressing microglia
Braak 2006 [140]	Autopsy 88 PD	—	—	—	MMSE 10% (range: 25–30) 16% (range: 21–24) 57% (range: 11–20) 17% (range: 0–10)	Braak'stages site: 1‐ DMNV 2‐ Medulla oblongata and Pontin tegmentum 3‐ Midbrain 4‐ Basal prosencephalon and mesocortex 5‐ Neocortex 6‐ Neocortex	PD Braak's Stage/no of patients 3/14 4/36 5/32 6/6 (> 80% MMSE 11–20)	No correlation with cognitive status
Dächsel 2007 [141]	Autopsy 9 PD 5 HC	78.1 ± 4.6 years	—	—	—	Visual cortex of the occipital lobe, putamen, amygdala, substantia nigra	Diminished α‐syn mRNA levels in substantia nigra (strongly), putamen, visual cortex. No change in amygdala	..
Hely 2008 [142]	Autopsy 4 PD 17 PDD	PD = 70 PDD = 79	—	—	—	Limbic/neocortex Brainstem	100% brainstem LB pathology consistent with the diagnosis of PD. Limbic and/or neocortical LBs prominent features in 8/17 with dementia	..
Beach 2009 [126]	Autopsy 87 HC 26 ILBD, 66 PD 40 DLB, 85 ADLB, 113 ADNLB 113	79.1 ± 6.9 years	46/20	10.6 ± 8.7	Last UPDRS III 41.0 ± 22.5 Last MMSE 20.3 ± 8.7	Olfactory bulb and tract, anterior medulla, anterior and mid‐pons, mid‐amygdala with adjacent transentorhinal area, anterior cingulate gyrus, middle temporal gyrus, middle frontal gyrus, inferior parietal lobule.	70%–90% in PD and DLB Most deposition in amygdala and cerebral cortex sections	Correlation with nigrostriatal degeneration

Beach 2010 [104]	Autopsy 23 HC, 7 ILBD 17 PD 9 DLB 19 ADLB 17 AD‐NLB	79.3 ± 7.5 years	13/4	—	Last MDS‐UPDRS III 30.1 ± 15.9 Last MMSE 26.5 ± 1.6	Spinal cord (C4‐5), (T6‐7), (L3‐4), (S1‐5) Middle cervical ganglia, middle ganglia of thoracic chain, vagus nerve	Spinal cord 96% Sympathetic ganglia 79% The vagus nerve 71% in PD and DLB	..
Quinn 2012 [143]	Autopsy 4 PD 8 DLB 5 AD 5 HC	79.5 ± 2.9	3/1	7.8	—	Middle temporal gyrus, cerebellum, superior parietal gyrus, superior frontal gyrus, superior occipital gyrus	Decreased soluble α‐syn in all the regions, with exception of the superior frontal gyrus No differences in α‐syn mRNA expression	..
Milber 2012 [131]	Autopsy 17 HC 33 ILBD 13 PD	Age at death PD 85.0 ± 1.11 ILBD 85.7 ± 0.90		8.3 ± 1.6		Ofactory bulb, medulla, pons, midbrain, hippocampus, amygdala, striatum at the level of the nucleus accumbens, basal forebrain, 7 neocortical regions (midfrontal, anterior superior and midtemporal, inferior parietal, calcarine, and primary motor/sensory gyri)	Prenigral ILBD cases with Braak PD stages 1 and 2 In PD nigral neuron density was observed to decrease as α‐Syn burden became more severe.	In ILBD nigral neuronal density seems to be independent of the regional distribution of LP deposition (a distinct process from PD?)
Borghammer 2022 [133]	Autopsy Vantaa dataset 27 early LBD+ Tokyo dataset 53 early LBD+	—	—	—	—	Sacral spinal cord, thoracic spinal cord, DMVN, LC, SN, amygdala, transentorhinal cortex, OB	Vantaa dataset LBs predominant in brainstem and PNS (*n* = 16, 2 in OB) LBs predominant in amygdala (*n* = 5, 5 in OB) Tokyo dataset LBs predominant in PNS (*n* = 9) LBs predominant in brainstem and PNS (*n* = 23, 2 in OB) LBs predominant in amygdala and PNS (*n* = 27, 24 in OB) LBs predominant in LC and/or SN (*n* = 3, 2 in OB)	..
Lin 2023 [134]	Autopsy 9 PD, 6 PDD 5 DLB 10 HC	All patients 80 ± 8	All patients 8/12	13.3 ± 6.8	—	SN (immunohistochemistry) SN and tracts between the SN and caudate nucleus, putamen, and DLPFC (MRI)	No significant differences between PD and PDD/DLB in LB density and LN in SN. Increased mean diffusivity of the SN‐DLPFC correlated with increased LN load in SN in the PD and PDD/DLB	Correlation among MRI‐measured alterations dopaminergic loss and LN load

Abbreviations: ADLB, Alzheimer's disease with Lewy bodies; ADNLB, Alzheimer's disease with no Lewy bodies; CDR, clinical dementia rating scale; DLB, dementia with Lewy bodies; DLPFC, dorsolateral prefrontal cortex; DMNV, dorsal motor nucleus of the vagus nerve; ILBD, incidental Lewy body disease; LC, locus coeruleus; MHC II, major histocompatibility complex class 2; NDD, neurodegenerative disorder clinically established; OB, olfactory bulb; PD, Parkinson's disease; PSP, progressive supranuclear pulsy; SN, substantia nigra; UPDRS, unified Parkinson's disease rating scale.

### 4.2 Peripheral α‐Syn Deposition and PD

Recent studies have focused on the evaluation of α‐syn deposition in peripheral tissues as a potential biomarker of PD. In particular, the presence of LBs and α‐syn deposits has been well‐documented in multiple peripheral tissues and organ nerves, including the retina, olfactory epithelium, sympathetic ganglia, enteric nervous system (ENS), salivary glands, cardiac sympathetic nerves, adrenal glands, pelvic ganglia, and skin [144, 145]. In addition, peripheral α‐syn pathology may precede widespread central disease and contribute to the prodromal symptoms of PD, such as dysautonomia, constipation, orthostatic hypotension, urinary and erectile dysfunction, and anosmia [146]. In this section, current data about α‐syn deposition in peripheral tissues, including skin, GI tract, and blood, are addressed and summarized in Tables 2, 3, 4.

**Table 2:** α‐Syn/LBs detection in the skin from PD patients.

Study	Number of patients/HC	Demographic information PD (mean or median ± SD or range)		Disease characteristics PD (mean or median ± SD or range)			Sites of investigation	α‐syn deposition (%) in PD patients	To note
		Age (years)	M/F	Disease duration (years)	MDS‐UPDRS III	Other			
Donadio 2020 [147]	25 PD 25 MSA‐P	74 ± 6.7	18/7	6.8 ± 4.1 years	—	LEDD 518 ± 250 mg	C7 Thigh leg	p‐syn deposits in the autonomic skin fibers of proximal and distal skin sites (100%)	..
Giannoccaro 2020 [148]	26 PD 13 PAF 7 DLB 13 MSA	71.7 ± 7.6	8/18	5.2 ± 4.8	27.7 ± 10.8	—	C7 Thigh leg	p‐syn deposits in skin autonomic nerves around annexes and/or around blood vessels (95.2%)	..
Liu X 2020 [149]	90 PD 30 HC	59.81 ± 9.15	43/47	3.98 ± 3.12	33.46 ± 20.07	—	C7 thigh distal leg forearm	p‐syn deposits in the single cervical site (83.3%)	..
Chahine 2020 [150]	59 PD 21 HC	63.1 ± 8.6 y.o.	41/18	57.7 ± 54.9 (months)	26.4 ± 11.9	LEDD 465 ± 429 mg	C7 Mid‐thigh	64.1%	..
Wang N 2020 [151]	29 PD 21 HC	66.3 ± 7.7	16/13	5.5 ± 5.1	29.4 ± 11.2	—	Thigh, distal leg, forearm	50 μm 100% 20 μm 90% 10 μm 73%	..
Manne 2020 [152]	37 PD 37 HC	—	—	—	—	—	Scalp	96% (by frozen samples) 75% (paraffin‐embedded samples)	..
Mazzetti 2020 [153]	57 PD 48 HC	61.6 ± 10.8	38/19	7.1 ± 5.6	19.3 ± 12.4	—	Forearm	α‐syn oligomers in synaptic terminals of autonomic fibers (89%)	..
Infante 2020 [154]	1 PD^OH+^ (three biopsies in 3 consecutive years)	67	1/0	At the first biopsy 10 months	I 15 II 25 III 31	LEDD I 0 II 200 III 350	C7 thigh distal leg	I 40% (deep dermis) 5% (autonomic nerve bundles) II 31% (adrenergic autonomic nerve) III 40% (adrenergic autonomic nerve terminals) 29% (muscle arrector pilorum)	..
Wang Z 2021 [155]	Autopsy 47 PD, 7 LBD 3 MSA, 17 AD, 8 PSP, 5 CBD 43 NNC Biopsy 20 PD, 20 NNC	Autopsy 80.1 ± 7.1 y.o. Biopsy 68.3 ± 7.3	Autopsy 34/13 Biopsy 12/8	—	—	—	Abdominal Scalp	95% (assessed by RT‐QuIC) 80% (assessed by PMCA)	..
Isonaka 2021 [156]	44 PD 16 HC 5 mutation carriers without PD	59.5 ± 13.9	21/23	11.5 ± 10.4	—	30 individuals with pathogenic mutation SNCA 3, PRKN 10, LRRK2 7, GBA 7, PARK7/DJ1 3	C2 region of the nape of the neck	95% PD 100% SNCA 100% LRRK2 83% GBA 0% biallelic PRKN	..
Giannoccaro 2022 [157]	26PD 18PSP, 8CBS 26 HC	71.7 ± 7.6 y.o.	8/18	5.2 ± 4.8	27.7 ± 10.8	—	C7 thigh distal leg	65% PD 7% PSP/CBS	..
Lama 2022 [158]	30 PD (15 with pain, 15 without pain) 30 HC	67.8 (range: 54–81)	23/7	11.1 (range: 0–22)	—	—	Lateral lower leg of individuals approximately 10 cm above the ankle	0% (α‐syn gene expression)	..
Nolano 2022 [159]	57 PD 43 MSA‐P	63.5 ± 9.4	36/21	16.8 ± 6.4 (months)	21.4 ± 5.2	NMSS 41.9 ± 34.8	Thigh Leg fingertip	96% PD 91% MSA	p‐syn deposits correlated positively with sudomotor function, epidermal, pilomotor and sudomotor nerve densities, and inversely with non‐motor symptoms and disease progression
Kuzkina 2023 [160]	39 PD 38 iRBD 23 HC	PD 65 (range: 50–79) iRBD 65.5 (range: 55–84)	PD 33/6 iRBD 33/5	8 (range: 1–22) years	21 (range: 2–47)	—	C7 Th10 Proximal leg	78.4% iRBD 70% PD 7.9% HC	No correlation between dermal α‐synuclein aggregation and duration of iRBD, RBDSQ, Non‐Motor Symptoms Questionnaire, MDS‐UPDRS‐III
Kuzkina 2023 [161]	27 PD 18 iRBD 6 atypical parkinsonism HC 30	PD 64.0 ± 8.7 RBD 65.9 ± 7.0	PD 20/7 iRBD 15/3	12.4 ± 5.6	—	—	Nasal brushing C7, thigh, leg	PD 55.5% Skin, 48.1% Nasal SAA iRBD 100 Skin %, 66.7% Nasal SAA	..

Abbreviations: AD, Alzheimer disease; ADLB, Alzheimer's disease with Lewy bodies; AD‐NLB, Alzheimer's disease with no Lewy bodies; CBD, corticobasal degeneration; DLB, dementia with Lewy body; iRBD, idiopathic REM sleep behavior disorders patients; LEDD, levo‐dopa equivalent dose; MSA‐P, multiple system atrophy parkinsonian type; MMSE, minimental state examination test; NNC, nonneurodegenerative control; PAF, pure autonomic failure; PD^OH+^, Parkinson's disease with orthostatic hypotension; PSP, progressive supranuclear palsy; PMCA, protein misfolding cyclic amplification; RT‐Quic, real‐time quaking‐induced conversion; SAA, seed amplification assays.

**Table 3:** α‐Syn/LBs detection in gastrointestinal tract from PD patients.

Study	Number of patients/HC	Demographic information PD (mean or median ± SD or range)		Disease characteristics PD (mean or median ± SD or range)				Sites of investigation	α‐Syn deposition (%) in PD patients	To note
		Age (years)	M/F	Disease duration (years)	MDS‐UPDRS III	MMSE	Other			
Braak 2006 [162]	Autopsy 5 PD 5 NNC	69.8 ± 5.9	3/2	—	—	—	—	Stomach	100% in neurons of the submucosal Meissner plexus, whose axons project into the gastric mucosa and terminate in direct proximity to fundic glands	..
Beach 2010 [104]	Autopsy 23 HC 7 ILBD 17 PD 9 DLB 19 ADLB 17 AD‐NLB	79.3 ± 7.5	13/4	—	30.1 ± 15.9	26.5 ± 1.6	—	Upper third and lower third of esophagus, stomach (body), duodenum, jejunum, ileum, transverse colon, sigmoid colon, rectum, submandibular gland, liver, pancreas (head), gall bladder	Submandibular Gland: 39% Esophagus: 33% Stomach: 22% Duodenum: 17% Jejunum: 8.3% Ileum: 7% Colon: 6% Rectum: 6%	Rostrocaudal gradient: submandibular gland and lower esophagus have the highest frequency of PASH, followed by the stomach, small bowel regions, large bowel regions and rectum. PASH more frequently present in the myenteric plexus than in the submucosal plexus
Del Tredici 2011 [145]	Autopsy 3 PD 1 ILBD	72.5 ± 9.3	1/3	14 ± 2.65	—	—	Case 1 Incidental LP experienced Gastrointestinal symptoms (primarly indigestion). Case 2 Developed dementia and hallucinations. Case 3 Experienced fatigue, shortness of breath, nocturia, and constipation. Developed dementia. Case 4 Developed cognitive decline and she became bedridden.	One or both submandibular glands, the superior cervical ganglion, one or both vagal nerves at the level of the bifurcation of the external and internal carotid arteries, the distal esophagus and transition zone to the gastric cardia, the celiac ganglion, the thyroid gland.	100% PD (mostly in esophagus, stomach, vagal nerve)	..
Annerino 2012 [163]	Autopsy 13 PD 4 incidental LBD 12 HC	PD 76.3 ± 6.6 iLBD 84.3 ± 8.4	PD 9/4 iLBD 1/3	—	—	—	—	Stomach duodenum ileum transverse colon rectum	100% PD‐ILB	No neuronal loss in the myenteric plexus in PD α‐syn was more frequent in the proximal than distal GI tract No correlation between gut pathology with brain pathology. < 3% of α‐syn aggregates were TH positive.
Gold 2013 [164]	Autopsy 10 PD 77 NNC 8 AD	77.0	—	—	—	—	—	Colon	100% PD 52% HC (without LB)	..
Devos D 2013 [165]	19 PD 14 HC	60.8 ± 8	11/8	9.9 ± 1.3	4.7 ± 0.7		LEDD 712 ± 151 mg Rome III score 3.5 ± 0.3	Ascending colon	33% PD	No significant difference in the expression of pro‐inflammatory cytokines or glial marker between patients with and without enteric Lewy pathology
Gelpi 2014 [103]	Autopsy 4 PD 6 PDD 5 DLB 13 non Lewy body disease	PD 79.8 ± 8.1 PDD 79.7 ± 6.1	PD 1/3 PDD 3/3	PD 11.5 ± 9.0 PDD 18 ± 8.8	—	—	—	Brain and spinal cord, pituitary gland, vagus nerve at brainstem and thoracic level, stellate ganglion and paravertebral sympathetic ganglia of both sides, mesenteric sympathetic ganglion, adrenal glands and surrounding fat tissue, distal esophagus, stomach (cardias, corpus, and fundus), ileum, colon (transverse and descending), rectum, periprostatic plexus or uterus, urinary bladder, heart, abdominal skin, and psoas muscle	pANS: 15/15 PD/DLB (stellate and sympathetic ganglia 100%; vagus nerve 86.7%; gastrointestinal tract 86.7%; adrenal gland and/or surrounding fat 53.3%; heart 100%; genitourinary tract 13.3%)	α‐syn aggregates was not related to the presence of inflammatory infiltrates
Hilton 2014 [85]	62 PD (at least 8 years priori the diagnosis) 161 HC	68 (range: 46–85)	42/20	—	—	—	PD α‐syn+ RBD 29% Constipation 86% Postural Hypotension 100% Cognitive impairment 86% PD α‐syn – RBD 22% Constipation 65% Postural Hypotension 29% Cognitive impairment 47%	Esophagus Stomach small intestine Large intestine Gall bladder	Esophagus 0% Stomach 8.6% Small intestine 13.3% Large intestine 13.2% Gall bladder 0%	All patients with α‐syn had autonomic symptoms. Some of the positive gastrointestinal biopsies showed inflammation
Mu 2015 [166]	Autopsy 10 PD 4 HC	74.5 ± 4.0	8/2	15.8 ± 9.0	42.2 ± 19.8	—	Dysphagia in 5/10 PD patients	Tongue, Pharynx, Larynx, upper esophagus	PD 100% (highest density in anterior tonsillar pillar, aryepiglottic fold, upper esophagus) HC 0%	PD patients with dysphagia had more p‐syn‐immunoreactive axons in the regions that are critical for initiating the swallowing reflex
Visanji 2015 [167]	22 PD 7 PD > 5 years of disease 11 HC	Early PD 64 (54–87) Later PD 62 (50–75)	Early PD 12/10 Later PD 2/5	Early PD 1 (0–3) Later PD 10 (5–16)	Early PD 14 (5–24) Later PD 14 (6–28)	—	Early PD LEDD 300 (0–600) mg Later PD LEDD 800 (100‐2770) mg	Sigmoid colon Rectum	80% early PD 100% later 100% HC	..
Beach 2016 [72]	Autopsy 5 PD 5 HC	77.4 ± 6.9	5 M	15.2 ± 8.0	36.8 ± 16.9	—	—	Sigmoid colon	100% PD	..
Chung SJ 2016 [168]	38 PD 13 MSA 53 HC	66.31 ± 8.25 y.o.	23/15	7.7 ± 4.7	23.42 ± 9.35	—	LEDD 820.4 ± 333.2 mg MMSE 26.16 ± 3.48 NMSS 50.92 ± 36.22	Stomach Ascending colon Trasverse colon Sigmoid colon	PD Stomach 36% Ascending‐Colon 8% Trasverse‐Colon 21% Sigmoid colon 0%	..
Lee HJ 2018 [169]	35 PD 52 HC	67 (range: 50–79)	15/20	7 (range: 2–21)	23.2 ± 9.5	—	LEDD 816 ± 339 mg	Fundus Antrum ascending colon transverse colon sigmoid colon	34% PD 38% HC	No correlation between the α‐syn pathology in ENS and gastrointestinal symptoms.
Fenyi 2019 [106]	18 PD 11 HC	63.7 ± 7.7	12/6	—	—	—	—	Rectum Sigmoid Antrum	55% PD2/2 upper GI biopsies 7/12 sigmoid biopsies 1/4 rectal biopsies 1/11 HC who developed later parkinsonian feature	..
Chahine 2020 [150]	59 PD 21 HC	63.1 ± 8.6	41/18	57.7 ± 54.9	26.4 ± 11.9	—	LEDD 465 ± 429 SCOPA‐AUT 12.5 ± 5.5	Sigmoid colon (also skin, submandibular gland)	14% PD	No correlation between intraindividual measures of α‐syn across the various tissues
Tanei 2021 [170]	Autopsy 518 older individuals. Between them: PD = 8 PDD/DLB = 38	LBP+ M = 81.1 ± 9.5 F = 86.4 ± 8.5	98/80	—	—	—	—	Brain, spinal cord, olfactory bulb and tract, the sympathetic ganglia, left heart ventricle, lower esophagus, adrenal gland. Skin of the upper arm and thigh	Lower esophagus 92.1% PDD 75% PD	43.8% of older individuals exhibited LP, of whom in the esophagus. LP of the esophagus significantly associated with autonomic failures and among PNS regions, correlated the most with LBD progression
Beach 2021 [171]	Autopsy 53 PD 111 NNC 33 ILBD	PD 79.1 ± 6.4	40/13	13.3 ± 7.3	38.8 ± 20.5	—	—	Stomach (body) and vagus nerve (adjacent to the carotid artery in the neck)	PD Stomach 81% vagus nerve 89%	No p‐syn in the vagus nerve or stomach of subjects without brain p‐syn

Abbreviations: ADLB, Alzheimer's disease with Lewy bodies; ADNLB, Alzheimer's disease with no Lewy bodies; DLB, dementia with Lewy bodies; DLPFC, dorsolateral prefrontal cortex; DMNV, dorsal motor nucleus of the vagus nerve; ILBD, incidental Lewy body disease; LEDD, levo‐dopa equivalent dose; LTS, Lewy‐type a‐synucleinopathy; MHC II, major histocompatibility complex class 2; MMSE, minimental state examination test; NDD, neurodegenerative disorder clinically established; OB, olfactory bulb; PASH, phosphorylated a‐synuclein histopathology; PD, Parkinson's disease; PSP, progressive supranuclear pulsy; SCOPA‐AUT, Scales for Outcomes in Parkinson's Disease‐Autonomic questionnaire; SN, substantia nigra.

**Table 4:** α‐Syn detection in plasma/serum from PD patients.

Study	Number of patients/HC	Demographic information (mean or median ± SD or range)		Demographic information (mean or median ± SD or range)				Thecnique for detecting α‐syn	Results
		Age (years)	M/F	Disease duration (years)	UPDRS III	H‐Y	MMSE		
Lee 2006 [172]	105 PD 38 MSA 51 HC	64.5 ± 11.4	45/60	44.6 ± 37.7 months	—	2.4 ± 0.9	—	ELISA	Significantly elevated levels in PD patients compared with MSA and controls
Li 2007 [173]	27 PD (13 EO, 14 LO) 11 HC	EOPD 62.8 ± 7.0 LOPD 71.9 ± 6.8	EOPD 5/8 LOPD 5/9	EOPD 18.8 ± 9.1 LOPD 9.6 ± 5.8		EOPD 2.8 ± 0.8 LOPD 2.4 ± 0.8		Quantitative Western Blot	Significant decrease in PD patients (especially in EOPD)
Duran 2010 [174]	53 ntPD 42 tPD 60 HC	ntPD 64.3 ± 1.6 tPD 65.4 ± 1.4	ntPD 25/28 tPD 14/28	tPD 9.2 ± 1.1 ntPD H‐Y	—	ntPD 1.5 ± 0.08tPD 2.56 ± 0.2	—	ELISA	Levels in the ntPD and tPD groups were similar, higher than in HC
Gorostidi 2012 [175]	134 PD32 PD LRRK2 109 HC	PD 69.0 ± 10.6 PD LRRK2 68.0 ± 10.1	PD 77/57 PD LRRK2 13/19	PD 6.2 ± 5.3 PD LRRK2 7.5 ± 6.3	—	PD 2.0 ± 0.8 PD LRRK2 2.1 ± 0.8	—	ELISA	Significant decrease in plasma total α‐syn levels PD
Caranci 2013 [176]	69 PD 110 HC	Age 64.6 ± 9.3	40/29	10.8 ± 7.3	median 17	—	—	ELISA	No difference between PD and HC
Foulds 2013 [177]	189 Newly diagnosticated PD	61.9 ± 9.7	119/70	5.1 ± 4.1		—	—	ELISA	Higher in PD compared to HC at baseline. t‐α‐Syn increases over time
Wang 2014 [178]	21 PD (13 FOG+) 15 HC	FOG+66.9 ± 6.7 FOG‐67.8 ± 4.2	FOG+8/5 FOG‐6/2	FOG+6.8 ± 3.8 FOG‐4.1 ± 1.6 years	FOG+31.5 ± 20.7 FOG‐33.6 ± 10.5	—	—	Luminex	Levels significantly decreased in FOG+ compared to controls
Shi 2014 [179]	267 PD 215 HC	66.3 ± 9.1	145/119	9.6 ± 6.6	28.4 ± 12.6	2.4 ± 0.7	28.0 ± 2.6	Antibody‐coated superparamagnetic microbeads	Exosomal α‐syn higher in PD than HC
Ishii 2015 [180]	30 PD 58 HC	Age 65.7 ± 9.7	18/12	3.9 ± 3.8 years	22.1 ± 9.9	—		ELISA with or without HAI	Significantly lower in the PD group than HC
Lin 2017 [181]	80 PD 34 HC	69.6 ± 12.3	60/54	7.5 y ± 5.2	17.4 ± 9.6 (on‐state)	—	24.3 ± 5.7	Immunomagnetic reduction	Significantly higher in patients with PD
NG 2019 [182]	170 PD 51 HC	66.6 ± 9.5	99/71	5.0 ± 5.0 years	24.8 ± 12.5		25.4 ± 3.7	SIMOA	Significantly higher in PD than controls
Bougea 2019 [183]	724 PD 569 no‐PD (meta‐anlysis)	—	—	—	—	—	—	Elisa, Luminex, immunomagnetic reduction‐based immunoassay	Levels significantly higher in PD patients than healthy controls
Si 2019 [184]	38 PD (22 Tremor dominant TD; 16 non tremor dominant NTD) 21 ET 28 HC	PD TD 62.68 ± 10.62 PD NTD 62.13 ± 10.61	PD TD 12/10 PD NTD 8/8	PD TD 19.18 ± 14.04 months PD NTD 35.81 ± 29.56 months	PD TD 18.27 ± 9.39 PD NTD 18.88 ± 10.94	PD TD 1.61 ± 0.60 PD NTD 1.72 ± 0.52		ExoQuick Dynabeads Antibody Coupling Kit Western blot‐ELISA	CNS‐derived exosomal α‐synuclein is reduced in PD compared to ET.
Jiang 2020 [185]	230 PD 45 PDD 65 RBD, 21 DLB, 14 MSA 65 FTD, 35 PSP, 45 CBS 144 HC	—	PD 149/81 PDD 34/11	—	—	—	—	Immunoblot Electrochemiluminescence	Increased α‐synuclein egress in serum neuronal exosomes precedes the diagnosis of PD, persists with disease progression and differentiates PD from atypical parkinsonism.
Chang 2020 [186]	48 PD 40 HC	67.2 ± 9.8	24/24	9.1 ± 6.5	—	2.8 ± 1.4	23.9 ± 5.8	Monoclonal antibodies with magnetic nanoparticles	Levels significantly higher in PD patients than HC
Shim 2020 [189]	20 PD 20 HC	71.4 ± 8.76	13/7	—	—	—	—	ELISA	No difference between PD and HC
Zhao 2020 [188]	30 PD 30 HC 28 MDD	69.8 (49‐81)	16/14	—	—	11 H‐Y1 16 H‐Y2 1 H‐Y3 2 H‐Y4	—	Immunoprecipitation‐mass spectrometry	PD and HC groups showed similar levels
Chen 2020 [189]	60 PD 28 HC	62.8 ± 9.1	21/39	Median 1 (0.0–3.0)	Median 20.0 (16.0–34.0)	—	Median 26 (22.0–27.0)	Immunomagnetic reduction‐based immunoassay	Higher plasma levels in PD compared to HC
Fan 2020 [190]	43 PD 24 HC	Age 58.4 ± 1.4	24/19	2.3 ± 0.3	31.4 ± 2.1	—		UPlex Assay	Higher plasma levels of α‐syn in PD compared to HC
Niu 2020 [191]	36 Early PD 17 advanced PD 20 RBD 21 HC	65 ± 5.3	25/47	—	22.3 ± 10.3	2.0 ± 0.5	27.6 ± 2.6	Western blot Electrochemiluminescence	α‐syn neuronal exosomes significantly higher in early stage PD patients compared with HCs and correlated with motor scores Longitudinally increased α‐syn is associated with higher progression of motor symptoms.
Zou 2020 [192]	93 PD 85 HC	66.93 ± 9.52	53/40	4.26 ± 2.52	28.73 ± 16.04	2.8 ± 0.5	24.28 ± 2.88	Dynabeads Antibody Ligation Kit Western blot Mass Spectrometry Analysis	Increased α‐syn concentrations in L1CAM exosomes in PD compared to HC.
Chung 2021 [193]	116 PD 46 HC	69.7 ± 8.4	62/54	2.82 ± 2.48	22.48 ± 9.85	—	24.17 ± 6.36	Immunomagnetic reduction–based immunoassay for EV α‐syn levels	Significantly lower levels of α‐syn in plasma EVs in PD patients than HC.
Youssef 2021 [194]	49 PD 48 HC	67 ± 1.2	32/17	4.0 ± 0.3	—	2.0 ± 0.1	—	ELISA (3 distinct kits)	Levels significantly increased in PD than HC with all the 3 kits
Agliardi 2021 [195]	32 PD 40 HC	69.47 ± 8.56	21/11	6.28 ± 3.63	28.52 ± 13.16	2 (1–3)	24.23 ± 2.48 (MoCA)	ExoQuick Western‐blot‐ELISA	Oligomeric exosomal α‐syn significantly augmented in neuronal derived extravescicles and positively correlated with disease duration and severity of PD.
Dutta 2021 [196]	50 PD 30 MSA 51 HC	71.5 ± 9.5	32/19	8.4 ± 5.0	25.4 ± 15.3	2.5 ± 1.0	26.5 ± 6.2	Immunoprecipitation Electrochemiluminescence ELISA	The ratio between α‐syn concentrations in oligodendroglial exosomes compared to neuronal exosomes is sensitive biomarker for distinguishing between PD and MSA
Zheng 2021 [197]	36 PD 36 HC	70.4 ± 0.64	19/17					Total Exosome Isolation kit (Invitrogen) Western Blot	α‐syn/total α‐syn and oligomeric p‐α‐syn/total p‐α‐syn ratio in plasma exosomes may be applied to assist the PD diagnosis
Kluge 2022 [80]	30 PD 50 non‐PD	67 (46–84)	21/9	4 (1–16)	25 ± 15	2 (1–5)		Immunoblot Western‐blot Seeding assay	Pathological α‐syn exosomal conformer detected in all PD patients
Okuzumi 2023 [198]	221 PD 39 MSA, 10 DLB, 9 RBD 128 NNC, 30 PSP, 25 AD, 17 PD PRKN	66 ± 10	98/123	6.9 ± 5.8	13 ± 10	2.1 ± 1.0		IP/RT‐QuIC	High diagnostic performance for differentiating PD versus controls

Abbreviations: DLB, dementia with lewy body patients; ELISA, enzyme‐linked immunosorbent assay; EO, early onset; ET, essential tremor patiennts; EV, extracellular vescicle; FOG, PD patients with freezing of gait; HA, heterophilic antibody inhibitor; HC, healthy control; H‐Y, Hoehn and Yahr scale; IP/RTQuIC, immunoprecipitation‐based real‐time quaking‐induced conversion; LO, late onset; MDD, major depressive disorder; MMSE, mini‐mental state examination; MoCA, Montreal cognitive assessment; MSA, multiple system atrophy; ntPD, non treated PD patients; PD, Parkinson's disease patients; PDD, Parkinson's disease with dementia patient; PD LRRK2, Parkinson disease with LRRK2 mutation carrier patient; RBD, REM sleep behavior disorder patient; SIMOA, single‐molecule array; tPD, treated PD patients; UPDRS, unified Parkinson's disease rating scale.

#### 4.2.1 α‐Syn Deposition in Skin

Hairy skin is characterized by a complex autonomic innervation pattern, including sympathetic adrenergic and cholinergic fibers in all dermal structures, few parasympathetic fibers in skin arterioles, and arrector pili muscles and sensory nerves found in all autonomic structures involved in reflex regulation mechanisms [199]. Morphological analysis has shown that total α‐syn is constitutively expressed within the synaptic terminals in skin biopsies from healthy subjects. In particular, total α‐syn has been detected in sweat glands, arrector pilorum muscles, and blood vessels from control subjects [200].

Using sampled skin from the chest and forearm of autopsied patients and an immunohistochemical approach, Ikemura et al. found LN in the sympathetic nerve fascicles of the dermis and subcutaneous tissue in 10 out of 14 PD patients [201]. These findings logically prompted several groups to study if the detection of pathological α‐syn by immunohistochemical means in the biopsied skin could be an interesting tool for the diagnosis of PD. Since 2010, more than 30 studies on this subject have been published, and detailing these studies one by one is definitely beyond the scope of the current review. However, the main findings of these studies can be summarized as follows: (i) Skin biopsies (performed at multiple sites and with optimized staining protocols) allow to detect α‐syn deposits and discriminate between PD and controls with acceptable sensitivity and an excellent specificity [202, 203]; (ii) skin biopsy of the cervical C7 paravertebral provide the best sensitivity for the detection of α‐syn deposits [204]; (iii) dermal α‐syn deposits is an early phenomenon as it can be observed in skin biopsies from patients with idiopathic REM sleep behavior disorder (iRBD) [205], a well‐established clinical non‐motor risk marker of PD. At first glance, all the above findings are rather convincing and strongly suggest that the detection of dermal pathological α‐syn can be used for the diagnosis of patients with either prodromal or overt PD. The interpretation of skin α‐syn staining is nevertheless delicate and alternative approaches to immunohistochemistry would be helpful. At present, the most promising approaches are based on ultrasensitive techniques such as protein misfolding cyclic amplification (PMCA) and real‐time quaking‐induced conversion (RT‐QuIC) assays, which have been recently shown to efficiently amplify small amounts of aggregated α‐syn in skin biopsies from iRBD subjects with excellent specificity and sensitivity (Table 2) [160, 206].

#### 4.2.2 α‐Syn Deposition in the GI Tract

Regarding the ENS, the presence of LBs and neurites in the GI tract was described almost 40 years ago in two seminal reports [207, 208]. Subsequently, Wakabayashi et al. found LBs in the GI tract of seven consecutive autopsies performed in PD patients [209], and more recently, Beach et al. reported LB and LN in the gut of 11 of 17 PD patients [104]. In both studies, Lewy pathology was distributed in both the myenteric and submucosal plexus from the upper esophagus to the rectum following a rostrocaudal gradient [104, 209]. Remarkably, when specific histochemical procedures were used (analysis of multiple slides of thick sections of the lower esophagus), α‐syn histopathology was found in 14 out of 15 PD patients, suggesting that the pathology is scattered but nearly constant in the ENS [104].

As with the skin, the accessibility of the ENS has led many groups to study the presence of α‐syn deposits using routine GI biopsies. Most of the existing studies, which used immunohistochemical staining of formalin‐fixed paraffin‐embedded samples, have had conflicting results regarding sensitivity and specificity [210, 211]. An alternative approach with microdissection of the biopsies to perform whole‐mount immunostaining has provided interesting results in terms of specificity and sensitivity, but this approach can hardly be used routinely [212, 213]. Again, just like for skin biopsies, PMCA and real‐time quaking‐induced conversion RT‐QuIC assays might be helpful to efficiently amplify small amounts of aggregated α‐syn from GI biopsies in PD subjects (Table 3) [106].

#### 4.2.3 α‐Syn Deposition in the Blood

α‐Syn is physiologically expressed in the blood cells, including erythrocytes and platelets (more than 99%) and peripheral blood mononuclear cells and plasma (less than 1%) [214]. However, the quantity of α‐syn in the blood can be influenced by red blood cells contamination and hemolysis. Indeed, a low degree of contamination could result in a substantially increased concentration in plasma and serum of α‐syn [215].

Over the last years, several studies have focused on the assessment of α‐syn deposition in the plasma, serum, and circulating EVs from PD patients, using different assays, including validated enzyme‐linked immunosorbent assay (ELISA) kits, single molecule arrays (SIMOA), Luminex assay, and immunomagnetic reduction assays or RT‐QuIC [173, 174, 182, 183, 198]. Most of the evidence has reported that total α‐syn is significantly increased in the plasma from PD patients when compared to healthy controls [172, 174, 177, 179, 181, 182, 183, 189, 190, 194, 196, 198], while others reported no differences or lower levels of circulating α‐syn in PD patients [173, 175, 176, 180, 187, 188, 193]. These conflicting findings could result from the employment of different assays, sample degradation, including hemolysis, and different clinical features of recruited patients, such as age, disease duration, and severity. However, most of recent studies have shown that the increased levels of α‐syn in the blood correlated with the severity of motor dysfunctions, poorer cognitive impairment, and with an increase in circulating inflammatory mediators [176, 181, 182, 186, 189, 190, 193].

Of interest, several studies in PD patients have focused on the evaluation of α‐syn species in blood‐derived EVs, regarded as membrane particles of cellular origin that accumulate pathological α‐syn forms and promote its spreading among cells [215]. EVs, including exosomes, microvesicles, and apoptotic bodies, are produced by almost all cell types and can be released in several body fluids, including plasma, serum, urine, and CSF [216]. In the setting of PD, most studies have evaluated EVs‐derived α‐syn, including exosomes and microvesicles released by neurons, in the blood [192, 197, 215]. In particular, Shi et al. reported that plasma neural‐derived exosomes (L1 cell adhesion molecule‐containing exosomes) from PD patients contain higher α‐syn levels as compared with control subjects HC, and this pattern correlated with disease severity [179]. Jiang et al. showed that neuron‐derived exosomal α‐syn was increased in iRBD and early PD patients as compared to multiple system atrophy (MSA), other neurodegenerative diseases individuals and controls, suggesting that exosomal α‐syn accumulation precedes PD diagnosis, persists with disease progression and in combination with clusterin predicts and differentiates PD from atypical parkinsonism [185]. Likewise, Dutta et al. tested and validated blood‐based α‐syn concentrations in neuronal and oligodendroglial exosomes isolated from serum or plasma of controls, PD, and MSA patients, and they observed that the assessment of α‐syn in oligodendroglial‐derived exosomes can discriminate among PD, controls and MSA subjects [196]. Zou et al. showed that that the increase in EVs‐derived α‐syn correlated positively with EVs‐derived Linc‐POU3F3, a long noncoding RNA involved in the pathophysiological events in PD and, negatively with lysosomal enzyme β‐glucocerebrosidase (GCase), activity whom reduction is associated with PD severity [192]. Of interest, increased oligomeric α‐syn forms levels were detected in circulating neuronal exosomes derived from PD patients, and this pattern was found to be correlated with a decrease in SNARE proteins, including syntaxin‐1A (STX‐1A) and synaptobrevin2 (VAMP‐2), and with disease duration and severity [195, 197]. Increased α‐syn levels in plasma neuronal exosomes were detected in early PD patients compared with HCs, and this pattern correlated with UPDRS III/(I + II + III), Non‐Motor Symptoms Questionnaire, and Sniffin’ sticks 16 item test scores. After a mean follow‐up of 22 months in early PD patients, increased plasma α‐syn levels were found to be associated with a higher risk for motor symptom progression. No significant differences in exosomal α‐syn levels were observed among iRBD patients and control subjects [191]. Of interest, a recent study, assessing α‐syn levels in neuronal and non‐neuronal‐derived EVs from blood, revealed that the detection and amplification of pathological soluble α‐syn conformers in plasma neuronal EVs is highly promising as a reliable pre‐mortem biomarker for PD [80, 191]. These findings provide new insights for the development of a standardized, non‐invasive assay aimed at detecting pathology‐associated α‐syn extracted from blood. Only one study reported that circulating exosomes‐derived α‐syn is reduced in PD as compared to essential tremor patients and healthy subjects [217]. This discrepancy could depend on different methodological approaches, including the extraction of exosomes from blood and the method of quantification of α‐syn.

### 4.3 Summary

Clinical evidence indicates that PD patients are characterized by α‐syn deposition in many regions of the CNS and in peripheral tissues, including skin, gut, blood, and circulating EVs. However, correlations between α‐syn accumulation with disease duration, severity, and motor and non‐motor symptoms are complex and variable. In particular, most studies in brain tissues seem to be quite dated, and, most importantly, since being from postmortem patients, they do not allow to understand α‐syn initial deposition, its spreading, and its role in neurodegeneration and neuroinflammation. The more recent studies aimed at evaluating α‐syn deposition in skin, gut, blood, and cirvulating EVs have better clarified the role of α‐syn pathology in PD. Indeed, increased α‐syn in the blood and plasma/serum EVs was found to correlate with PD severity and symptoms. In addition, an interesting study has shown that the increased circulating levels of α‐syn correlated with IL‐1β levels, suggesting that α‐syn could contribute to immune/inflammatory responses in PD. However, several issues remain to be clarified. For instance, the exact initiation and spreading of α‐syn pathology remains debated, and most importantly, evidence about the effects of pathological α‐syn forms in neuroinflammatory and neurodegenerative processes in PD needs to be further investigated. Moreover, it remains to be determined whether the evaluation of the initial localization of pathological α‐syn forms by several assays, including immunolabeling, RT‐QuIC, or validated ELISA kits, can represent a useful diagnostic and prognostic biomarker of PD. In this context, validated assays to evaluate α‐syn deposits in several tissues from PD patients still need to be implemented.

## 5 Disease‐Modifying Pharmacological Strategies Targeting Directly or Indirectly α‐syn in PD

The involvement of α‐syn in neuroinflammation and neurodegeneration associated with PD is fostering research on the potential therapeutic benefits resulting from the direct or indirect pharmacological blockade of α‐syn. At present, several studies aimed at investigating the pharmacological role of α‐syn in PD, have been performed in in vivo and in vitro experimental models as well as in patients. In this section, the available preclinical and clinical studies testing the effects of the direct and indirect blockade of α‐syn are discussed and summarized in Tables 5 and 6.

**Table 5:** Effects of pharmacological compounds targeting directly or indirectly α‐synuclein in animal models of PD.

Therapeutic approach (category)	Drug/compound	Mouse model of PD	Therapeutic effect	Study
Targeting directly α‐syn				
Reducing α‐syn production with ASOs or RNAi	ASOs or RNAi infusion	• transgenic PD mice overexpressing human α‐syn • rat and non‐human primate lentivirus model of PD	↓ α‐syn expression in hippocampus, cortex and substance nigra	Alacron‐Aris 2018 [218], Sapru MK 2006 [219], Lewis J 2008 [220], McCormack AL 2010 [221], Gorbatyuk OS 2010 [222]
Inhibition of α‐syn aggregation and aggregate reduction	HSP70 and HSP104 Anle138b Nanobodies (VH14*PEST and NbSyn87)	• A30P • rat lentivirus model of PD • A30P • Rotenone mouse model of PD • rats over‐expressing wild‐type α‐syn	↓ phosphorylated α‐syn inclusions in nigrostriatal dopaminergic neurons ↓ α‐syn oligomer accumulation; counteract neuronal degeneration and disease progression remove α‐syn aggregates, replenish striatal dopamine and improve motor function	Lo Bianco C 2008 [223], Moloney TC 2014 [224], Klucken J 2004 [225] Wagner J 2013 [226] Brundin 2017 [227]
Increasing degradation of extracellular α‐synuclein: active and passive immunization	PRX002 MEDI1341 BAN0805	• Transgenic PD mice overexpressing human α‐syn • A53T	↓ α‐syn accumulation along with an improvement in synaptic functions and motor/memory abilities	Games D 2014 [228], Schofield 2019 [229], Nordström E 2021 [230]
Targeting indirectly α‐syn				
Increasing GCase activity	Ambroxol hydrochloride	• Transgenic PD mice overexpressing human α‐syn	↓ total and phosphorylated α‐syn in different brain regions	Migdalska‐Richards A 2016 [231]
Inhibiting c‐Abl activity	Nilotinib	• A53T	↑ autophagic clearance of α‐syn and prevent dopaminergic neural death from α‐syn toxicity	Crunkhorn S. 2023 [232], L. Hebron 2013 [233]
Increasing degradation of intracellular α‐syn aggregates	MSDC‐0160	• MPTP • α‐syn‐based *Caenorhabditis elegans* model • En1^+/−^	↓ α‐syn aggregates and ↓neuroinflammatory processes	Ghosh A 2016 [234]

*Note:* ↓ reduction; ↑ increase.

Abbreviations: α‐syn, α‐synuclein; MPTP, 1‐methyl‐ 4‐phenyl‐1, 2, 3, 6‐tetrahydropyridine; PD, Parkinson's disease.

**Table 6:** Current disease‐modifying pharmacological strategies aimed at counteracting α‐synuclein.

Therapeutic approach (category)	Drug/compound	Target/action	Study type	Clinicaltrial.gov identifier
Passive immunization against α‐syn	RO7046015/PRX002 (prasinezumab), a humanized IgG1 monoclonal antibody (intravenous administration)	α‐syn C‐terminal antibody	Phase 2b recruiting patients with early PD	NCT03100149 (PASADENA) active with results [235]
			Phase 2b recruiting patients with early PD	NCT04777331(PADOVA)
	TAK‐341 or MEDI1341, humanized IgG1 monoclonal antibody(intravenous administration)	α‐syn C‐terminal antibody	Phase 2 recruiting patients with early MSA	NCT05526391
	Lu AF82422, humanized IgG1 monoclonal antibody (intravenous administration)	α‐syn C‐terminal antibody	Phase 2 recruiting patients with early MSA	NCT05104476
Active immunization against α‐syn	UB‐312 (intramuscular injection)	Synthetic peptide vaccine inducing antibodies that against specifically oligomeric and fibrillar α‐syn	Phase 1b, recruiting patients with PD and MSA	NCT05634876
	ACI‐7104 (intramuscular injection)	Optimized vaccine formulation of the PD01A	Phase 2, recruiting early PD patients	NCT06015841 (VacSYn)
Decreasing α‐syn production	BIIB094 (intrathecal injection)	ASO drug targeting the mRNA of LRRK2	Phase 1, recruiting PD patients with or without variations in the LRRK2 gene	NCT03976349 (REASON)
	ION464 or BIIB101, (intrathecal injection)	ASO drug targeting the SNCA gene	Phase 1, recruiting MSA patients	NCT04165486 (HORIZON)
Inhibition α‐syn Aggregation	UCB0599 (minzasolmin), oral administration	α‐syn misfolding inhibitor	Phase 1, recruiting healthy participants	NCT05845645
			Phase 2, enrolling by invitation new onset PD patients in early‐start versus delayed‐start	NCT05543252
			Phase 2, recruiting early PD patients	NCT04658186
	BIIB122 (oral administration)	Inhibitor of LRRK2	Phase 2b, recruiting early PD patients	NCT05348785 (LUMA)
	Radotinib (oral administration)	BCR‐ABL1 tyrosine kinase inhibitor	Phase 2, recruiting PD patients	NCT04691661
	Vodobatinib or K0706 (oral administration)	BCR‐ABL1 tyrosine kinase inhibitor	Phase 2, recruiting early PD patients	NCT03655236 (PROSEEK)
	Risvodetinib or IkT‐148009 (oral administration)	BCR‐ABL1 tyrosine kinase inhibitor	Phase 2, recruiting untreated PD patients	NCT05424276
Regulation α‐syn gene expression	CST‐103, co‐administered with CST‐107 (oral administration)	β2‐AR agonist (clenbuterol)	Phase 2, recruiting 4 subject populations with Neurodegenerative Disorders – PD with (RBD) and Depressive Symptoms – MCI with Depressive Symptoms – DLB with Cognitive Fluctuations – PDD with Cognitive Fluctuations	NCT04739423
	CST‐2032, administered with CST‐107 (oral administration)	β2‐AR agonist	Phase 2, recruiting patients with MCI or dementia Due to PD or AD	NCT05104463

Abbreviations: α‐syn, α‐synuclein; AD, Alzheimer's Disease; ASO, antisense oligonucleotide; β2‐AR, beta2 adrenoceptor; DLB, Dementia with Lewy Bodies; LRRK2, leucine rich repeat kinase 2; MCI, mild cognitive impairment; MSA, multiple system atrophy; RBD, rapid eye movement (REM) sleep behavior disorder; PD, Parkinson's disease; PDD, Parkinson's Disease Dementia.

### 5.1 Pre‐Clinical Evidence

a. **Direct targeting of α‐syn**
  i. *Decrease in α‐syn expression*. With antisense oligonucleotides (ASOs) or with RNA interference (RNAi). In particular, studies in transgenic and lentivirus murine and non‐human primate models of PD have documented a decrease of α‐syn expression in the substantia nigra, hippocampus, and cortical regions after infusion with ASOs, small hairpin (shRNA) and short interfering (siRNA) [218, 219, 220, 221, 222];
  ii. *Inhibition of α‐syn aggregation and/or aggregate reduction*. Among these pharmacological strategies, two molecular chaperones, HSP70 and HSP104, have been found to decrease α‐syn aggregates both in in vitro nigrostriatal dopaminergic neurons obtained from transgenic mice as well as in different brain regions of rat PD models [223, 224, 225]. Another similar approach is represented by a class of small molecules recently conceived to specifically target oligomeric forms of α‐syn (NPT100‐18A, NPT200‐11, and Anle138b), orally bioavailable and brain penetrant [236, 237]. In particular, Anle138b is one of the few compounds successfully tested in several in vivo PD mouse models with promising results [226], while the other small molecules mentioned above have been tested only in in vitro dopaminergic neuronal cell cultures. Accordingly, further studies on animal models are strongly required to corroborate the therapeutic effects of these molecules.
    Of interest, over the last years, nanobodies (small antibody fragments that target antigens intracellularly) have been developed. Among them, nanobody VH14*PEST and nanobody NbSyn87 resulted effective in decreasing α‐syn aggregates, replenishing striatal dopamine, and improving motor functions in rats overexpressing wild‐type α‐syn [227]. However, despite these promising results, a significant challenge is to maintain high levels of the nanobodies in specific brain regions for prolonged periods and, therefore, current drug delivery technology would require direct delivery to affected regions using viral vectors;
  iii. *Increasing degradation of extracellular α‐syn: active and passive immunization*. Several studies reported that both active immunization (stimulation of the immune system to produce antibodies against toxic α‐syn forms) and passive immunization (using anti‐α‐syn antibodies) have been found to promote neuroprotective effects in transgenic models of PD [238, 239, 240]. In particular, the administration of PRX002 (humanized monoclonal antibody— Prothena/Roche), MEDI1341 (antibody targeting extracellular α‐syn through passive immunization—Astra Zeneca/Takeda), and BAN0805 (humanized mAb targeting α‐syn oligos and protofibrils) decreased central α‐syn accumulation along with an improvement in synaptic functions and motor/memory abilities in transgenic and lentivirus mouse model of PD [228, 229, 230].

b. **Indirect targeting of α‐syn**
  iv. *Increasing GCase activity*. Mutations of GCase (an enzyme involved in glycolipid metabolism) or a decrease in its activity have been reported to contribute to α‐syn misfolding in several synucleinopathies, including PD [231]. Ambroxol hydrochloride, a small molecule able to enhance GCase activity and consequently increase α‐syn clearance, has been demonstrated to decrease the total and phosphorylated α‐syn in different brain regions of transgenic PD mice [241], thus suggesting that the indirect targeting of α‐syn using GCase activators could represent a promising therapeutic strategy;
  v. *Inhibiting c‐Abl activity*. A hyperactivation of c‐Abelson (Abl) tyrosine kinase has been reported to contribute to α‐syn aggregation [232]. Nilotinib, a drug approved for the treatment of imatinib‐resistant chronic myelogenous leukemia, has been shown to increase autophagic clearance of α‐syn and counteract dopaminergic neural death in brain tissues from transgenic A53T mouse [232, 242]. However, no further studies on other pre‐clinical PD models have been performed, and, therefore, intensive research efforts are strongly needed;
  vi. *Increasing degradation of intracellular α‐syn aggregates*. Rapamycin and its analogs have been shown to promote the clearance of pathological α‐syn aggregates via autophagic processes. However, these compounds show limited utility since they lack specificity, also acting on other signaling pathways involved in immunosuppression; therefore, this feature limits their use to PD patients where long‐term treatment would be necessary. Accordingly, researchers have investigated other compounds that are more selective and able to promote autophagy. Among them, the compound MSDC‐0160 showed to exert a neuroprotective effect on midbrain and nigral dopaminergic neurons along with a decrease in α‐syn aggregates and neuroinflammatory processes in toxin‐induced PD mice, α‐syn‐based *Caenorhabditis elegans* model and En1^+/−^ (genetic model) [234], suggesting that the pharmacological modulation of autophagy could represent an intriguing strategy to counteract α‐syn‐mediated neuroinflammation and neurodegeneration associated with PD. However, further studies are strongly required to better clarify the putative beneficial effect of MSDC‐0160 on the intracellular aggregated α‐syn isoforms.

### 5.2 Clinical Trials

i. *Increasing degradation of brain extracellular α‐syn: active and passive immunization*. At present, five monoclonal antibodies (PRX002, BIIB054, ABVV‐0805, MEDI1341, LU AF82422) have been tested or are currently being evaluated in Phases 1–3 clinical trials in patients with synucleinopathies. The PRX002/prasinezumab (a humanized version of the C‐terminal antibody 9E4) and BIIB054/Cimpanemab (NI‐202, a fully human‐derived monoclonal antibody directed against the N‐terminal epitope of α‐syn) were recently evaluated in two similar multicenter, randomized, double‐blinded trials (the PASADENA—NCT03100149—and the SPARK study—NCT03318523—, respectively) [235, 243]. In these studies, early‐stage PD patients (Hoehn and Yahr stage < 3) with an abnormal dopamine transporter (DaT) scan have been recruited. In this trial, neither the primary nor secondary endpoints, represented by a reduction in the MDS‐UPDRS scale (Movement Disorder Society‐Sponsored Revision of the Unified Parkinson's Disease Rating Scale) total score and in DaT imaging over a 52‐week period, respectively, were met, except for a trend of slower motor progression with the lower dose of PRX002 in the PASADENA study that led to the launch of a Phase 2b study (PADOVA—NCT04777331) [235, 243, 244]. Another humanized monoclonal antibody tested in a clinical trial was ABBV‐0805 (BAN0805, selective for soluble aggregated α‐syn) has been discontinued for strategic reasons although reported a favorable tolerability profile (NCT04127695) [245]. Likewise, MEDI1341 (TAK‐341) and LU AF82422, both monoclonal antibodies targeting the C‐terminal of α‐syn [229], showed good tolerance in both healthy subject and PD patients in completed Phase 1 clinical trials (NCT03272165 and NCT04449484 for MEDI1341; NCT03611569 for LU AF82422) [246, 247].
  Active immunization approaches with synthetic peptides mimicking human α‐syn have also been tested in PD patients in Phase 1 clinical trials, and they have shown a good safety and tolerability profile [248, 249]. In particular, the vaccine UB‐312 is currently in Phase 1 clinical trial in PD patients (NCT05634876) [250, 251], but no data are currently available. The molecules Affitope PD01A and PD03A targeting α‐syn aggregates have shown to be safe, well‐tolerated and highly immunogenic in two Phase 1 trials conducted in early PD (NCT01568099 for PD01A and NCT02267434 for PD03A) [252, 253, 254]; thus, an optimized version of the vaccine PD01A, now called ACI‐7104, is entered into a Phase 2 trial (NCT06015841).
ii. *Decrease in α‐syn production*. A Phase 1 trial (REASON, NCT03976349) is currently ongoing in patients with or without a PD‐related *LRRK2* mutation treated with ASO intrathecal administration [255]. In particular, in this study, BIIB094 will be tested for its ability to reduce the LRRK2 expression and, consequently, the α‐syn fibril formation [255]. In this regard, it is noteworthy that the complete gene inactivation of α‐syn can trigger detrimental effects on neuronal and non‐neuronal cells; therefore, special attention is required to avoid an excessive reduction of physiological α‐syn levels [256]. An alternative approach to decrease α‐syn expression seeks to interfere with the transcription of α‐syn gene [257, 258, 259, 260]. In particular, Mittal et al. demonstrated that the activation of β2‐adrenoreceptors (β2‐AR) reduced *SNCA* expression by regulating α‐syn transcription, thus suggesting a potential neuroprotective effect of β2‐AR. In addition, the company CuraSen Therapeutics Inc. has reported positive results from a Phase 2 clinical trial (NCT04739423) in patients with neurodegenerative conditions (PD with Rapid Eye Movement Sleep Behavior Disorder or dementia, Dementia with LBs and Mild cognitive impairment) by using a combination of β2‐AR agonists: clenbuterol (CST‐103) and nadolol (CST‐107) [261].
iii. *Inhibition of α‐syn aggregation and aggregate reduction*. Recently, the brain‐penetrant molecule UCB0599 (minzasolmin), an enantiomer of NPT‐200‐11, moved into two Phase 1 studies (NCT05845645 and NCT04875962) to test safety/tolerability and pharmacokinetics profile in healthy participants [262]. Based on the promising results, UCB0599 is underway in the Phase 2 clinical trials (NCT04658186 and NCT05543252). Another similar promising small molecule called Anle138b has reached the clinical state of development, completing Phase 1 trials in healthy participants (NCT04208152) and patients with mild to moderate PD (NCT04685265) [263].
  Of note, molecules able to target LRRK2 are emerging as a potential therapeutical strategy to reduce α‐syn pathology through the restoration of lysosomal functions [264]. In this regard, the selective LRRK2 inhibitor, DNL201, recently completed the Phase 1 clinical trial (NCT04551534) and 1b studies (NCT03710707) showing a dose‐dependent impact on lysosomal function with an acceptable safety profile [265].
iv. *Inhibiting c‐Abl activity*. Nilotinib is able to inhibit c‐Abl (increased in PD patients) [266] and DDR1/2 (discoidin domain receptors), thus promoting autophagic clearance of pathological α‐syn [267]. Although preliminary studies showed acceptable safety and tolerability in PD patients along with favorable effects on CSF biomarkers, including brain dopamine turnover, oligomeric α‐syn, and hyperphosphorylated tau [268, 269], Nilotinib demonstrated poor penetration in the brain and no relevant symptomatic benefit on measures of disability in a successive multi‐dose placebo‐controlled Phase 2 study (NCT03205488) [270]; consequently, further clinical trial developments are currently lacking. Other Abl‐tyrosine kinase inhibitors with better CNS penetration (Radotinib, ikt‐148009 or Risvodetinib and Vodobatinib or K0706) are currently underway in Phase 2 multicentre trials (NCT04691661, NCT05424276, and NCT03655236, respectively).

### 5.3 Summary

Pre‐clinical studies targeting directly or indirectly pathological α‐syn have provided promising results. Among them, immunotherapy and small molecules able to dissociate α‐syn aggregates have raised a significant interest as potential therapeutical approaches to halt or slow down the progression of PD. Unfortunately, the results deriving from clinical trials have not shown promising results as compared to the pre‐clinical ones. Indeed, most drugs targeting α‐syn have been found to be safe and well tolerated, they have shown poor efficacy in halting or slowing down PD progression as well as in alleviating motor and non‐motor symptoms. Encouraging results have emerged only from the PASADENA trial, showing a trend of slower motor progression in early PD patients that led to the launch of a Phase 2b study.

In summary, based on current findings, some important issues remain to be discussed: (1) the degeneration of nigral neurons can occur before α‐syn deposition, and, most importantly, it remains controversial the timing of α‐syn accumulation in the pathogenic cascade of PD; (2) the physiological functions of α‐syn in neuronal and non‐neuronal cells as well as the exact nature of disease‐relevant species (e.g., oligomers vs fibrils) needs still to be clarified; (3) the recruitment of PD patients, since most of the clinical trials include early symptomatic PD patients with already advanced nigrostriatal dopaminergic neurodegeneration; (4) poor blood–brain barrier penetration of drugs; (5) α‐syn targeting approaches may require time to produce any observable effects. Based on these considerations, in vivo markers of α‐syn pathology in humans able to measure target engagement should be identified. In addition, clinical studies in very early patients with a high risk of developing PD, including iRBD subjects, and the presence of pathological α‐syn deposition in gut, skin, or blood should be performed to clarify whether targeting α‐syn can represent the best pharmacological strategy for the treatment of PD.

## 6 Conclusions and Future Perspectives

Several studies have shown that pathological α‐syn forms in the brain and in the gut can accumulate in neuronal and non‐neuronal cells and induce neuronal degeneration and neuroinflammatory responses, respectively. In parallel, pathological α‐syn can be secreted in extracellular space and, in turn, it can activate neighboring neuronal and non‐neuronal cells as well as recruit peripheral immune/inflammatory cells [271], thus creating a sort of vicious circle that contributes to the ongoing neuroinflammatory and neurodegenerative processes. In this setting, it is conceivable that α‐syn acts as a bridging plug across central and systemic neuroinflammation and neurodegeneration in PD. However, the pathological forms of α‐syn mainly involved in α‐syn spreading and PD pathology remain to be clarified. Available data from clinical studies, though suggest that the evaluation of α‐syn especially in the blood and circulating EVs can represent a biomarker of PD, in terms of disease severity and symptoms and systemic inflammation, but does not allow to establish the causal relationship between α‐syn and brain pathology and, most importantly, whether its accumulation precedes or represents a mere consequence of PD.

Regarding its pharmacological relevance, α‐syn could indeed represent a promising therapeutic target for the treatment of PD. However, the clinical trials conducted so far have mostly recruited patients already diagnosed with PD, where neurodegenerative and neuroinflammatory processes have likely already been triggered, potentially impacting the effectiveness of such targeted therapies.

Thus, further clinical and experimental studies in humans, including early, iRBD, non‐movement disorder PD patients, and in animal models are needed to better clarify the pathophysiological and pharmacological role of α‐syn deposition in PD onset and progression as well as its relevance as diagnostic or prognostic marker of PD.

## Author Contributions

Gabriele Bellini, Carolina Pellegrini, and Pascal Derkinderen: conceptualization. Gabriele Bellini, Carolina Pellegrini, Giovanni Palermo, Vanessa D'Antongiovanni, Matteo Fornai: writing the original draft. Pellegrini Carolina, Luca Antonioli, Pascal Derkinderen, Nunzia Bernardini, Roberto Ceravolo: supervision, writing, review and editing. All authors read and approved the final manuscript.

## Conflicts of Interest

The authors declare no conflicts of interest.

## Data Availability

No new data were created or analyzed in this study. Data sharing is not applicable to this article. All the literature used for this review is listed in the bibliography.
